# Sleep and olfactory cortical plasticity

**DOI:** 10.3389/fnbeh.2014.00134

**Published:** 2014-04-22

**Authors:** Dylan C. Barnes, Donald A. Wilson

**Affiliations:** ^1^Emotional Brain Institute, Nathan Kline Institute for Psychiatric ResearchOrangeburg, NY, USA; ^2^Behavioral and Cognitive Neuroscience Program, City University of New YorkNew York, NY, USA; ^3^Department of Biology, University of OklahomaNorman, OK, USA; ^4^Department of Child and Adolescent Psychiatry, New York University Langone School of MedicineNew York, NY, USA

**Keywords:** olfaction, piriform cortex, slow-wave sleep, odor memory, odor perception, memory consolidation

## Abstract

In many systems, sleep plays a vital role in memory consolidation and synaptic homeostasis. These processes together help store information of biological significance and reset synaptic circuits to facilitate acquisition of information in the future. In this review, we describe recent evidence of sleep-dependent changes in olfactory system structure and function which contribute to odor memory and perception. During slow-wave sleep, the piriform cortex becomes hypo-responsive to odor stimulation and instead displays sharp-wave activity similar to that observed within the hippocampal formation. Furthermore, the functional connectivity between the piriform cortex and other cortical and limbic regions is enhanced during slow-wave sleep compared to waking. This combination of conditions may allow odor memory consolidation to occur during a state of reduced external interference and facilitate association of odor memories with stored hedonic and contextual cues. Evidence consistent with sleep-dependent odor replay within olfactory cortical circuits is presented. These data suggest that both the strength and precision of odor memories is sleep-dependent. The work further emphasizes the critical role of synaptic plasticity and memory in not only odor memory but also basic odor perception. The work also suggests a possible link between sleep disturbances that are frequently co-morbid with a wide range of pathologies including Alzheimer’s disease, schizophrenia and depression and the known olfactory impairments associated with those disorders.

## Introduction

**Figure 1:**
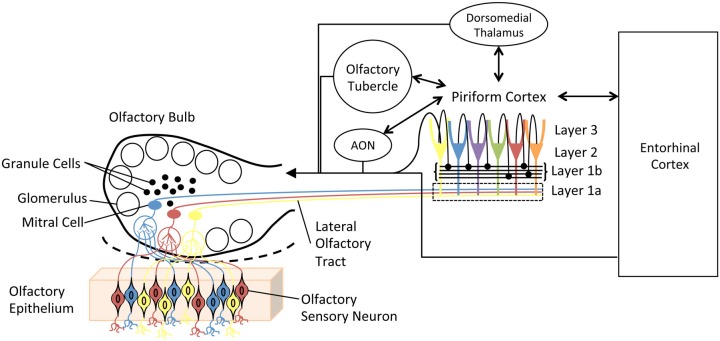
**Simplified schematic diagram of the main olfactory system**. Olfactory sensory neurons in the olfactory epithelium express one of a very large set of olfactory receptor genes. Here, cells expressing different genes are colored differently. Sensory neurons expressing the same gene converge onto glomeruli in the olfactory bulb (OB), where they synapse onto second order neurons, mitral and tufted cells. Individual mitral cells receive excitatory input from a single glomerulus, and thus received input from a homogeneous population of sensory neurons. Excitability of, and lateral interactions between, mitral cells are mediated in part by OB granule cells. Granule cells undergo continual neurogenesis and replacement throughout life. Furthermore, they are a primary target of descending inputs from olfactory cortical areas. The primary olfactory cortex (piriform cortex) functions as an combinatorial, auto-associative array, allowing convergence of input conveyed from different olfactory sensory neurons. This allows merging of odorant features into odor objects. In addition to merging odorant features, the piriform cortex also has extensive, reciprocal connections with a variety of limbic and cortical areas.

The olfactory system (Figure 1) is remarkably plastic. This experience-dependent plasticity is not reserved for higher-order olfactory cortical areas or zones of multisensory integration, but rather is expressed throughout the olfactory pathway from the nose to cortex. For example, in the olfactory epithelium, odor experience and associative training can modify olfactory sensory neuron receptor gene expression, survival and axonal targeting (Tyler et al., 2007; Jones et al., 2008; Tian and Ma, 2008; Kass et al., 2013). In the olfactory bulb (OB), such experience can modify OB glomerular size, number of juxtaglomerular neurons and glomerular responses (Fletcher, 2012; Miura et al., 2012). In addition, second-order neuron (mitral/tufted cell) structure (Gheusi et al., 2000; Davison and Ehlers, 2011), sensory physiology and excitability (Moreno et al., 2009; Boland and Alloy, 2013; Tononi and Cirelli, 2014) have also been shown to be sensitive to odor experience. Not only are OB principal neurons changed, but also the large population of OB inhibitory interneurons, granule cells undergo extreme odor experience-dependent changes in survival and physiology (Killgore and McBride, 2006; Moreno et al., 2009; Yokoyama et al., 2011). These learned changes in OB structure lead to changes in OB function, often measured as changes in local circuit oscillations (Grajski and Freeman, 1989; Martin et al., 2006; Chapuis et al., 2009; Kay and Beshel, 2010). Beyond the OB, odor experience has been shown to modify piriform cortical pyramidal cell synaptic and sensory physiology (Mouly et al., 2001; Wilson, 2003, 2010; Roesch et al., 2007; Cohen et al., 2008; Chapuis and Wilson, 2011; Saar et al., 2012), membrane excitability (Saar and Barkai, 2003) and dendritic structure (Knafo et al., 2004), as well as piriform cortical local circuit oscillations (Martin et al., 2006; Kay and Beshel, 2010; Chapuis and Wilson, 2011). Finally, functional connectivity between the OB and olfactory cortex (Martin et al., 2006), and between these primary olfactory structures and higher-order cortical areas such as the hippocampus (Martin et al., 2007) and orbitofrontal cortex (Cohen et al., 2008) are also highly experience-dependent. This list is not meant to be exhaustive of all possible identified changes, but rather meant to exemplify the extent and diversity of changes induced by either passive odor exposure (or lack thereof) or associative conditioning.

Together, these experience-induced changes can modify olfactory system sensitivity and acuity, as well as link odor quality to associative meaning or hedonic valence. The changes can last as short as a few seconds, in the case of short-term odor adaptation (Best and Wilson, 2004) to as long as days, weeks or years in the case of associative memory (Fillion and Blass, 1986) or following long-term odor-exposure (Dalton and Wysocki, 1996). Apparently the most stable feature of the olfactory system is its constant change. Thus, as described elsewhere (Wilson and Stevenson, 2003; Wilson and Sullivan, 2011), olfactory perception lies at the interface between memory and sensation. That is, experience-dependent plasticity is required not only to allow the associative memory that strawberry aroma signals a pleasant, energy dense fruit, but also to allow the implicit memory underlying perception of the complex mixture of odorants emanating from the fruit as a unique odor object which English-speaking humans label “strawberry”.

Most forms of long-term memory require a process of consolidation, wherein temporary traces of encoded information become more permanently stored through post-experience modulation (McGaugh, 2013). Post-experience consolidation is in essence a “save now” process. Not all experiences need be permanently stored. Those events or stimuli that signaled something biologically significant (e.g., a source of food, safety or danger, or mating opportunity) may be more likely to be consolidated than other events. Thus for example, events that elevate peripheral epinephrine or central norepinephrine are more strongly consolidated than those that do not (McGaugh, 2013).

However, it is increasingly apparent that memory consolidation can also involve a sleep-dependent stage. Sleeping within a few hours of learning a new skill or new information enhances memory for that skill or information, compared to staying awake after the initial learning. This consolidation enhancement (enhanced long-term memory) can occur for motor skill memories, declarative/episodic memories, emotional memories and sensory memories/perceptual learning (Stickgold, 2005; Diekelmann and Born, 2010; Rasch and Born, 2013; Tononi and Cirelli, 2014).

Recent work has begun exploring the role of sleep in olfactory memory. Olfaction is interesting in this regard given the unusual anatomy of the olfactory pathway compared to all other sensory systems, most notably the relatively limited involvement of a thalamic nucleus prior to the primary sensory cortex. Two major approaches have been used in this research. First, odors have been used as contextual cues while some other task was ongoing, such as declarative or emotional memory task (Rasch et al., 2007; Eschenko et al., 2008; Hauner et al., 2013). This research has primarily utilized the odor context to manipulate sleep-dependent memory consolidation of these other forms of information (Rasch et al., 2007; Diekelmann et al., 2012; Hauner et al., 2013). Several recent reviews that include this work have been published (Rasch and Born, 2013; Shanahan and Gottfried, 2014) and this work will not be the focus of this paper. The second approach has been to focus on odor memory and the olfactory system itself. This work has examined state-dependent changes in olfactory system activity and connectivity, and explored how sleep directly modifies the olfactory system after odor experience. Based on this work, we argue here that olfactory system structure and function, and thus olfactory perception itself, is shaped in an odor-specific manner during post-odor experience sleep. These modifications are critical to both the associative meaning of learned odors, as well as the acuity of odor perception and memory. The work suggests that odor perception not only depends on the odors we smell, but also on the odors of which we dream.

## Sleep

Sleep or sleep-like states appear to occur in all animals, including invertebrates and vertebrates. Sleep is generally defined as a period of behavioral dormancy, though is not necessarily associated with a quiescent nervous system. In fact, the mammalian brain is highly active during sleep and undergoes shifts between several different sleep-related physiological states that together are referred to as sleep structure. Although sleep-related activity can occur unilaterally in marine mammals and some birds, generally the entire brain enters specific stages of sleep nearly simultaneously.

### Sleep structure

**Figure 2:**
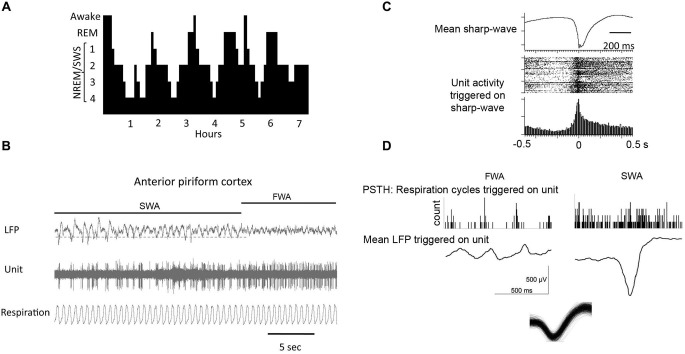
**(A)** During a typical human sleep bout early stages of the sleep bout are dominated by slow-wave sleep, while REM sleep becomes more prominent later in the bout. The same is true in rodents. **(B)** local field potential (LFP) recordings in piriform cortex showing spontaneous shifts between slow-wave and fast-wave activity. **(C)** During slow-wave sleep, piriform cortical single-units fire in phase with the sharp-waves as shown by the raster plot and peri-stimulus time histogram. **(D)** Piriform cortical single-unit and LFP activity are decoupled from respiration during slow-wave sleep compared to waking. Histograms show respiratory events as a function of single-unit spiking. LFP’s are mean waveforms triggered on single-unit spikes. Overdrawn waveform of single-unit used for analysis in D in shown at bottom.

In non-human mammals, there are two widely accepted behavioral phases of sleep: rapid eye movement (REM) or paradoxical sleep and slow wave sleep (SWS), also known as non-paradoxical or NREM sleep (Figure 2). In the circadian rhythm of most mammals, early nocturnal and diurnal sleep contains mostly SWS and late sleep is largely devoted to REM sleep (Tobler, 1995). Both of these sleep stages are characterized by specific field potential oscillations of brain activity. REM sleep is characterized by fast, low-amplitude oscillatory activity in the θ-band (4–8 Hz) and higher frequency bands characteristic of waking (van der Helm et al., 2011). REM sleep also includes ponto-geniculo-occipital (PGO) waves that are intense bursts of synchronized activity propagating from the pontine region of the brain stem to the lateral geniculate nucleus and visual cortex (Datta and O’Malley, 2013). Behavioral hallmarks of REM sleep are phasic bouts of REM (hence the phase’s name) and muscle atonia (Jones, 1979).

The predominant oscillatory activity in SWS is in the δ-band (0.5–4.0 Hz), which includes the less than 1 Hz slow oscillation (Buzsáki, 1986; Mölle et al., 2002; Montgomery et al., 2008). These oscillations are comprised of alternating periods of membrane depolarization, the “up” states, and hyperpolarization, “down” states. Most cortical neurons engage in slow oscillations and the firing patterns produce high synchrony across cellular populations in different brain regions (Amzica and Steriade, 1998; Steriade et al., 2001; Volgushev et al., 2006). The widespread synchrony of neuronal activity during SWS is the backbone of the slow oscillation and allows for a relatively global time scale in which activity is limited to the depolarized up states and terminated with the hyperpolarized down states (Luczak et al., 2007; Mölle and Born, 2011). Early sleep SWS is classically thought of as important for processing declarative and hippocampal based memories (Wagner and Born, 2008), although recent studies may point to complementary, rather than separate processes for memory consolidation (see below) (Rasch and Born, 2013).

A special type of oscillation seen in the hippocampus (Eschenko et al., 2008) and the piriform cortex (Wilson, 2010; Manabe et al., 2011; Narikiyo et al., 2014) during periods of quiet wakefulness and, more predominantly during SWS, is the sharp-wave ripple (SPW-R). These are fast depolarizing waves that were first seen in the CA3 region of the hippocampus and are superimposed by high frequency (100–300 Hz) ripple oscillations (Diekelmann and Born, 2010). During sharp-wave ripple events, small subpopulations of pyramidal cells that were active during prior wakefulness spontaneously fire in the same pattern as during learning (Pavlides and Winson, 1989) in a much shorter amount of time as compared to their original timing during encoding. The number of sharp-wave ripple events during post-learning sleep is also significantly correlated with the formation and strength of memories (Axmacher et al., 2008; Ramadan et al., 2009). The olfactory system is special in relation to other sensory systems as sharp-waves have also been described within the main olfactory cortex, the piriform cortex (Wilson and Yan, 2010; Manabe et al., 2011; Narikiyo et al., 2014) that are similar to those described in the hippocampus (Buzsáki, 1986), and that these sharp-waves appear to be generated independent of hippocampal origination, perhaps originating in the endopiriform nucleus (Behan and Haberly, 1999).

### Sleep is implicated in synaptic homeostasis

SWS is traditionally thought of as the marker for homeostatically regulated sleep pressure. After prolonged periods of wakefulness, sleep pressure is greatest as evidenced by a shorter latency to enter SWS following sleep onset and an increased power of slow wave activity (SWA), which gradually decreases from early to late sleep (Borbély, 1982; Borbély and Achermann, 1999; Riedner et al., 2007). However, in addition to sleep homeostasis, SWS may also play an important role in the homeostasis of synaptic weight. That is, sleep may be a time when the brain can re-adjust synaptic weights according to their recent history of use, returning them to a state where they can be maximally efficient at encoding new information—avoiding saturation and incapable of further strengthening (Tononi and Cirelli, 2003). This hypothesis of synaptic homeostasis allows for a widespread decrease in the strength of synaptic connections that take place during SWS. Plasticity-related gene expression follows a circadian rhythm and sleep deprivation causes a decrease in the expression of these genes (Guzman-Marin et al., 2006). During wakefulness, and especially during learning, there is a buildup of molecular correlates associated with long-term potentiation (LTP) such as BDNF and cAMP response element-binding protein (Silva, 2003). During SWS, however, the expression of these genes is significantly reduced or downscaled (Tononi and Cirelli, 2003). LTP also involves an increase in dendritic spine growth. Sleep, however, seems to act as a pruning mechanism during which time there is a decrease in the number of spines (Bushey et al., 2011). Synaptic downscaling, therefore, prevents saturation of the synaptic weight and reduces place and energy demands of a neural network, thereby allowing the system to reset to prepare for the encoding of new information during succeeding wakefulness (Horn et al., 1998a,b). The hypothesis promotes the consolidation of stronger memories, however, that were encoded during the pre-sleep period to remain and replay spontaneously while more trivial synapses can be reset. This active system consolidation implicates a certain selectivity of which memories to consolidate. It is likely that the widespread consolidation of everything recently learned would produce a system overflow. In fact, sleep does not benefit all memories, although the mechanisms that determine which memory will be tagged for consolidation and which will be forgotten during sleep is still unclear.

## Sleep and perceptual learning in thalamocortical systems

As noted above, sleep-dependent consolidation has been demonstrated for a variety of forms of information including declarative/episodic, motor, emotional and sensory memory. The prevailing view is that during sleep, recently acquired information is replayed, both within local neural networks (e.g., hippocampus or sensory cortex) and across networks (e.g., linking hippocampal and neocortical networks). This replay allows recently strengthened synapses to solidify those changes either biochemically or structurally, while other less critical synapses are reset to basal strengths, ready for plasticity another day (Stickgold, 2005; Cirelli and Tononi, 2008; Rasch and Born, 2013; Tononi and Cirelli, 2014). Performing replay during sleep, especially SWS, may be ideal as it is a period of hypo-responsiveness to external sensory inputs, and thus replay may be less prone to external interference.

Although sleep-dependent consolidation influences most forms of memory, here we focus on the perceptual learning to highlight evidence of sleep-dependent consolidation within thalamocortical sensory systems. Perceptual learning is an enhancement in perceptual acuity or sensitivity induced by training. Perceptual learning is associated with sensory system plasticity and is remarkably specific. For example, learning to make very precise judgments of the alignment of two vertical lines (e.g., a vernier scale) does not transfer to judgments of diagonal or horizontal lines. Similar selective improvements can be made in the other thalamocortical senses with appropriate training. The learned enhancement in sensory acuity is associated with narrowed receptive fields in sensory neocortex and/or enlargement of the sensory cortical region devoted to that segment of the sensory world (e.g., Recanzone et al., 1992; Godde et al., 1996; Kilgard and Merzenich, 1998; Xerri et al., 1999). The improvement in sensory acuity following perceptual learning is facilitated by sleep (Karni et al., 1994; Allen, 2003; Fenn et al., 2003; Atienza et al., 2004; Gottselig et al., 2004; Censor et al., 2006; Yotsumoto et al., 2009), although see Hussain et al. (2008) and Aberg et al. (2009) for counter–examples. Both REM (Karni et al., 1994) and SWS (Aeschbach et al., 2008) have been implicated in consolidation of perceptual learning. As noted above, SWS may be a very effective period for sleep-dependent replay of learned stimuli given that the sensory evoked activity in the thalamus becomes highly variable and reduced during SWS (McCormick and Bal, 1994; Steriade et al., 2001). This shift away from thalamic monitoring of external events may reduce interference during memory replay.

In a variety of paradigms, sleep structure during the post-training period is modified, with prolonged bouts of either REM or SWS, depending on the specific task (Tononi and Cirelli, 2014). This prolongation of sleep bouts is hypothesized to reflect the additional pressure required for memory storage. In addition, perceptual learning can influence sleep related activity in neocortical sensory systems in other ways. First, SWS related activity (EEG oscillation power or fMRI activation) is enhanced in sensory cortex during sleep after perceptual learning (Cantero et al., 2002; Yotsumoto et al., 2009; Bang et al., 2013), and specifically in those regions of sensory neocortex encoding the learned stimulus (Yotsumoto et al., 2009). Second, while slow waves are cortical-wide events generally driven by thalamocortical oscillations, recent evidence suggests that following visual perceptual learning, slow-waves may be preferentially initiated in primary visual cortex (Mascetti et al., 2013).

Together, these changes in sleep structure, and sleep-related oscillatory activity within the primary sensory system may promote consolidation and refinement of newly learned sensory representations, allowing enhanced perceptual acuity after sleep compared to a similar period of waking (Karni et al., 1994; Mednick et al., 2002; Allen, 2003; Fenn et al., 2003; Aeschbach et al., 2008). However, neocortical sensory systems evolved well after the primary olfactory system. Can the mammalian olfactory system, with a very different structure and relationship to thalamus, also support sleep-dependent memory consolidation?

## Sleep and olfactory system physiology

Sleep-dependent changes in neocortical function are largely shaped by changes in thalamic activity (Steriade et al., 2001; Buzsaki, 2006). For example, slow-waves derive from a synchronization of slow, δ-frequency oscillations generated in thalamic and neocortical networks. During this thalamocortical slow-wave activity, sensory evoked activity in the thalamus is reduced and more variable, resulting in reduced sensory cortical activation. In contrast, although there is an olfactory thalamic nucleus that contributes to odor processing and memory (Lu and Slotnick, 1990; Courtiol and Wilson, 2014), it is downstream of the primary olfactory cortex, not between the cortex and the periphery. Thus, the state-dependent gating performed by the thalamus in thalamocortical systems is missing in olfaction. Furthermore, rather than displaying slow-wave activity during SWS as in thalamocortical systems, the olfactory cortex (piriform cortex) displays sharp-wave ripples, similar to that observed in the hippocampal formation. Nonetheless, important parallels exist between the olfactory cortex and thalamocortical systems in sleep-wake state dependent physiology.

Although there is no thalamic state-dependent gate between the nose and the piriform cortex, the piriform cortex shows robust state-dependent fluctuations in odor responsiveness. During SWS, both piriform cortical single-units (Murakami et al., 2005; Wilson, 2010) and local field potentials (Barnes et al., 2011) show greatly attenuated odor responses. This SWS-dependent modulation is expressed both in unanesthetized animals spontaneously cycling between waking and SWS (Barnes et al., 2011; Manabe et al., 2011), and in urethane-anesthetized animals (Murakami et al., 2005; Wilson, 2010) that also show spontaneous fast-wave/slow-wave cycling. It should be emphasized that cortical odor-evoked activity is not completely eliminated during SWS, but is greatly reduced. In unanesthetized animals, odor responses appear unaffected by REM sleep states (Barnes et al., 2011). The human olfactory system appears similarly depressed during SWS (Carskadon and Herz, 2004).

During SWS, while the piriform cortex responsivity to the outside world is reduced, its activity shifts to sharp-wave/ripple like activity, reminiscent of that observed in the hippocampal formation. However, the piriform cortical sharp-wave activity is relatively independent of that observed simultaneously in the hippocampus (Manabe et al., 2011). Although the generator of these large cortical sharpwaves in unknown, one hypothesis is that they are driven by the highly auto-excitatory endopiriform nucleus, which has broad excitatory connections throughout piriform cortex (Behan and Haberly, 1999). In fact, the current sink for piriform cortical sharp-waves is located in layers II/III which is consistent with a intracortical association fiber/endopiriform driven potential (Wilson, 2010; Manabe et al., 2011). Piriform cortical layer II/III single-unit activity during these sharpwaves coincides with the deep recorded negative peak (Wilson, 2010; Manabe et al., 2011; Narikiyo et al., 2014). That is, units shift from primarily firing in phase with the respiratory cycle during waking or fast-wave states to firing en masse in phase with the sharpwaves during SWS.

This coherent firing of large piriform cortical pyramidal cell ensembles in phase with sharpwave evokes strong responses in monosynaptic targets of the piriform, including feedback to the OB (Manabe et al., 2011; Narikiyo et al., 2014). Functional connectivity/coherence of piriform cortex with limbic structures such as the basolateral amygdala and dorsal hippocampus, as well as neocortical areas is significantly enhanced during SWS compared to fast-wave states (Wilson and Yan, 2010; Wilson et al., 2011). Combined with the reduced response to sensory afferents, these changes in functional connectivity suggest a turning inward, perhaps consistent with the needs of replay and strengthening odor associations with meaning and hedonics.

One contributor to the state-dependent shift in piriform cortical activity is a change in neuromodulatory tone over the sleep-wake cycle, particularly acetylcholine (ACh). ACh plays a major role in odor processing and plasticity throughout the olfactory pathway from the OB (Ravel et al., 1994; Tsuno et al., 2008; Chaudhury et al., 2009), to olfactory cortex (Barkai et al., 1994; Wilson, 2001; Chapuis and Wilson, 2013). In the piriform cortex, ACh muscarinic receptor (AChmR) activation suppresses association fiber synaptic efficacy through a reduction in pre-synaptic glutamate release, with minimal effects on afferent fiber synapses from mitral cells (Hasselmo and Bower, 1992). This means that during waking or vigilance, when ACh levels are high, the piriform cortex would be strongly driven by afferent input from the bulb, while intracortical association fibers would be suppressed. During SWS however, when ACh levels drop, the intracortical association fiber system would be released from cholinergic suppression and could more effectively influence cortical activity. Such intracortical excitation between recently activated piriform cortical pyramidal cells during SWS may be important in replay of recently experienced odors.

## Sleep and odor memory

As noted above, a number of groups have used odors and odor contexts as cues in declarative, procedural or emotional memory tasks (Rasch et al., 2007; Eschenko et al., 2008; Diekelmann et al., 2011; Arzi et al., 2012; Hauner et al., 2013). This work has emphasized how odors delivered during sleep can modulate consolidation of various forms of memory in limbic or other brain regions. Here, we focus specifically on work examining how sleep related activity shapes the olfactory system itself, in turn shaping odor coding, perception and memory.

### Odor experience affects sleep structure

Laboratory housed rats and mice spend approximately 40% of a 24 h day in SWS (Barnes et al., 2011). Left alone for a 4 h period during the light cycle, rodents enter SWS within 5–15 min and then cycle between SWS, REM sleep and waking, with REM sleep bouts emerging after the initial SWS bouts. As is true in a variety of conditioning tasks, during a 4 h period following odor-fear conditioning rats spend significantly more time in SWS than control rats, as recorded within the piriform cortex (Barnes et al., 2011; Barnes and Wilson, 2014). In our paradigm, there was a slight, non-significant decrease in REM sleep, thus the total duration of sleep did not change (Barnes et al., 2011). Importantly, the percentage increase in post-training SWS duration strongly correlated with the strength of fear memory 24 h later (Barnes et al., 2011).

Thus, during SWS, the primary olfactory cortex is hypo-responsive to environmental stimulation, instead displaying sharp-wave activity, and following fear conditioning, animals spend more time in this state. It is hypothesized that the piriform cortical sharpwave activity during SWS may contribute to olfactory system reorganization and odor memory consolidation during a time of reduced interference from new odor stimulation.

### Odor replay modulates memory strength and precision

As an initial examination of whether odors are replayed in the piriform cortex during sharp-wave activity, we asked whether the temporal structure of single-unit activity during sharp-waves is affected by recent (15–30 min) odor experience that occurred during fast-wave state (Wilson, 2010). Piriform cortical single-units were recorded in anesthetized rats, which, though surgically anesthetized, naturally cycle between fast-wave and slow-wave states (Fontanini and Bower, 2005; Murakami et al., 2005; Wilson, 2010). Single-unit activity during piriform cortical sharpwaves was characterized and then the animal was allowed to spontaneously shift to fastwave state. During fastwave state, the animal was repeatedly exposed to an odor to which the recorded unit responded, or in control animals to no odor or to an odor to which the cell did not respond. After the stimulation, the animal was allowed to spontaneously return to slow-wave state, and again unit activity was characterized relative to sharpwave events. In cells that had been stimulated with odor during the fast-wave state, the temporal structure of sharp-wave related unit activity shifted and became more variable, compared to either control group (Wilson, 2010). Thus, the temporal structure of piriform cortical single-unit activity during slow-wave state was shaped by past odor experience. These data do not confirm that the cells were replaying the odor during a sharpwave, but are consistent with that interpretation. Unfortunately spontaneous single-unit activity in the piriform cortex is very slow (Roesch et al., 2007; Chen et al., 2011), thus some of the analytical techniques to discern actual “replay” that are used in other brain regions are currently less effective here. To address this problem, we decided to impose replay directly following odor fear conditioning.

Odor fear conditioning can induce either highly odor-specific fear responses (e.g., freezing) or more generalized odor-evoked freezing, depending on the nature of the training protocol (Chen et al., 2011). Differential conditioning, involving both a CS+ (predicts footshock) and a CS− (does not predict footshock) induces fear responses selectively to the CS+. Conditioning with a CS+ but no CS−, in contrast, induces generalized fear responses to a wide range of odors. These different behavioral outcomes are associated with distinct changes in piriform cortical single-unit odor coding (Chen et al., 2011). Differential conditioning, which induces odor specific fear, results in a narrowing of piriform cortical single-unit odor receptive fields compared to pseudo-conditioned controls, i.e., an enhancement in odor acuity. In contrast, conditioning without a CS−, which results in generalized odor fear, is associated with a broadening of piriform cortical single-unit odor receptive fields, i.e., a reduction in acuity. This suggests that the precision of the odor memory is at least in part due to changes in piriform cortical odor coding. Similar results can be observed after appetitive conditioning (Chapuis and Wilson, 2011). Does cortical activity during post-training sleep contribute to the strength and/or precision of this odor memory?

To test this, we utilized olfactomimetic electrical stimulation of the OB (Mouly et al., 2001) as the CS+ and CS− in a differential fear conditioning paradigm, with different spatial patterns of electrical stimulation serving as the different stimuli. We chose this type of stimulus because it allowed us to deliver identical stimuli, with precise temporal control during training and different post-training states, regardless of the animal’s posture or respiration in the different states. We further hypothesized that the strength of the direct OB stimulation would allow activation of the piriform cortex regardless of behavioral state.

Rats were trained using these olfactomimetic stimuli in a differential fear conditioning task, and then placed in a quiet chamber for the 4 h post-training. Piriform cortical local field potentials and neck muscle EMG were recorded to identify sleep/wake states. Animals that were trained and then left alone during the 4 h post-training period displayed CS+ specific freezing the following day. However, the strength and accuracy of this memory could be modulated by post-training CS+ imposed replay (Barnes and Wilson, 2014). For example, animals that received imposed post-training replay (i.e., OB stimulation identical to the CS+ delivered during training) while they were awake showed reduced CS+ evoked freezing the following day, consistent with extinction. In contrast, animals that received the identical imposed replay selectively during slow-wave sleep showed a significant enhancement in the strength of the CS+ evoked response. This enhanced memory was selective for the CS+. Importantly, imposing replay during slow-wave sleep of a novel olfactomimetic stimulus not previously encountered, while the animal was hypothetically spontaneously replaying the learned stimulus resulted in normal memory strength for the CS+, but now the memory was generalized to all olfactomimetic stimuli tested (Barnes and Wilson, 2014).

We hypothesize that during post-training slow-wave sleep, ensembles of piriform cortical neurons that had been recently co-activated during training, preferentially fire together during piriform cortical sharp-waves in a form of spontaneous replay. This co-activation is facilitated by a release of intracortical association fiber synapse suppression due to low levels of cholinergic activity during slow-wave sleep (Hasselmo and Bower, 1992; Hasselmo and McGaughy, 2004). The co-activation during sharp-waves should strengthen excitatory synapses between neurons within the ensemble, enhancing representation of the learned odor (Linster et al., 2003, 2009). Adding noise to the replay by imposing the novel stimulation during slow-wave sleep should expand the membership of the ensemble to include irrelevant neurons, thus degrading the precision of the odor representation and inducing generalized fear responses.

If this model is correct, then disrupting intracortical association fiber synapses during the post-training period should impair odor memory strength and/or memory precision. To test this, we infused the GABA_B_ receptor agonist baclofen bilaterally into the piriform cortex during the post-training period. Baclofen selectively suppresses association fiber synapses in the piriform cortex (Tang and Hasselmo, 1994; Poo and Isaacson, 2011; Barnes and Wilson, 2014), and suppresses sharp-wave amplitude (Barnes and Wilson, 2014). Animals with suppressed association fiber synapses during the 4 h post-training period showed normal freezing to the CS+, however the freezing strongly generalized across odors. Thus, the post-training association fiber activity, presumably during spontaneous sleep-dependent replay, is necessary for odor memory precision the following day (Barnes and Wilson, 2014).

Together, this work suggests that piriform cortical sharp-wave activity during post-training slow-wave sleep allows for a strengthening of ensemble representation of the learned odor. This post-training replay can modify both the strength and the precision of the odor memory, at least in part via plasticity within the piriform cortex itself. However, it must be noted that co-activation of large piriform cortical ensembles during sharp-waves also result in propagation of spike trains out of the cortex to its monosynaptic neighbors. Thus, this provides an opportunity for piriform cortical manipulation not only of intra-cortical synapses, but also of its efferent targets during slow-wave sleep. These “downstream” effects have been the focus of recent work in Kensaku Mori’s group (Manabe et al., 2011; Yokoyama et al., 2011; Yamaguchi et al., 2013; Narikiyo et al., 2014).

### Odor replay modulates adult-born olfactory bulb neuron survival

One important role for odor learning is for memory of novel flavors. The majority of the perception of flavor is derived from volatile food odorants delivered to the olfactory epithelium via retronasal smell. While humans are believed to have the most advanced retronasal olfactory abilities (Shepherd, 2012), rodents also experience retronasal smell (Chapuis et al., 2009). In omnivore’s such as mus musculus and rattus norvegicus, learning about food odors, and their nutritional and/or illness producing characteristics is critical for survival. Interestingly, a common behavioral response in many mammals following a satiating meal is drowsiness and sleep; referred to a post-prandial sleep. For example, 50% or more of food deprived mice begin resting or fall asleep within 1 h of being given access to food (Yokoyama et al., 2011). Could post-prandial sleep contribute to memory for the odors and flavors of the consumed food?

As noted above, odor memory is associated with changes throughout the olfactory pathway. Perhaps the most extreme neural correlate of odor memory is differential survival of OB granule cells. OB granule cells are inhibitory interneurons which modulate the excitability of OB mitral and tufted cells and are the primary target of descending inputs from olfactory cortex. Importantly, granule cells display adult neurogenesis (Bayer, 1983), with survival of adult born neurons dependent on olfactory stimulation and activity (Killgore and McBride, 2006; Moreno et al., 2009). Differential experience-dependent granule cell survival may contribute to olfactory acuity and information storage (Gheusi et al., 2013). Yokoyama et al. (2011) have demonstrated that there is enhanced granule cell death in the few hours post-feeding. The extent of cell death is correlated with the amount of time spent in slow-wave sleep (though not REM sleep) (Yokoyama et al., 2011). Granule cell death is enhanced even more if the bulb is odor deprived during the food exposure. This suggests that those granule cells not activated by the food odors are selectively targeted for apoptosis during post-prandial sleep.

Mori’s group suggests that the strong descending sharpwave-associated pyramidal cell spiking from the olfactory cortex to the OB during slow-wave sleep (Manabe et al., 2011) may be the critical signal differentiating granule cell death and survival. Thus, it is hypothesized that during waking odor exposure, for example during the meal, granule cells activated by the odors are tagged by descending piriform cortical axonal input. Those cells not activated by the odor do not receive the same tagging. During subsequent post-prandial slow-wave sleep, cortical sharp-wave evoked descending activity, perhaps in concert with sleep-associated neuromodulatory inputs initiate cascades leading to non-tagged granule cell apoptosis (Yokoyama et al., 2011). Similar events may occur following other odor learning experiences, for example the fear conditioning protocols described above.

### Summary of major effects of sleep on olfactory cortex

**Figure 3:**
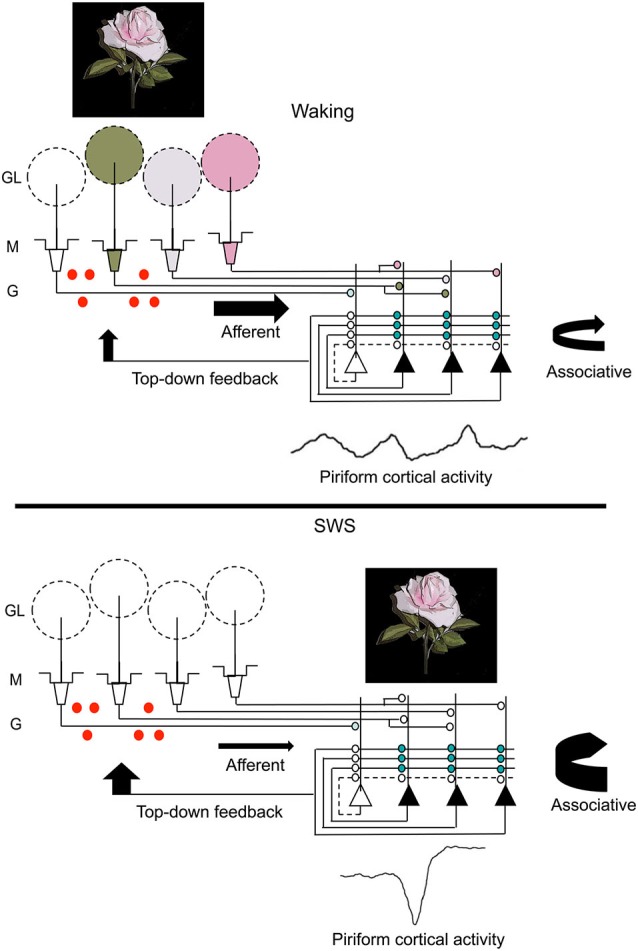
**A schematic representation of changes in olfactory system activity between waking and slow-wave sleep**. Odor stimulation during waking (symbolized by the rose) evokes odor-specific patterns of activity in the OB and mitral/tufted cell output to the piriform cortex. This afferent activity is respiration entrained and evokes intra-cortical association fiber activity linking co-active piriform cortical neurons. It also results in piriform cortical output, including feedback to OB granule cells (red dots) as well as to other regions of olfactory cortex and non-olfactory regions. During slow-wave sleep, the balance of afferent and intracortical activity shifts, with decreases in sensory-evoked input to piriform cortex and enhanced intra-cortical mediated activity, primarily during sharp-wave events. The sharp-wave associated activity replays cortical ensemble activity evoked by odors during waking. These strong, synchronous sharp-wave events help strengthen synaptic connections within odor-coding ensembles, as well as help shape OB granule cell survival in an odor-specific manner. Abbreviations: GL = glomerular layer, M = mitral cell layer, G = granule cell layer.

Combining the findings from these two paradigms leads to the suggestion that post-odor exposure slow-wave sleep contributes to changes across the olfactory system that contribute to both the strength and precision of odor memory (Figure 3). Slow-wave sleep associated piriform cortical sharp-waves allow strengthening of associations within cortical ensembles encoding specific odors in a replay-like manner. Reducing this association or imposing noise during replay impairs the precision of the odor memory. Sharp-wave evoked piriform cortical activity also induces strong activation of cortical efferent targets, such as the OB and perhaps other mono-synaptic targets (Courtiol and Wilson, 2014; Narikiyo et al., 2014), contributing to memory-associated changes in those structures.

## Sleep, pathology and odor perception

Olfactory deficits are associated with a variety of disorders including, but not limited to Alzheimer’s Disease (Murphy, 1999), Parkinson’s disease (Doty, 2012), schizophrenia (Malaspina et al., 2012) and major depression (van Mill et al., 2010). All of these disorders are also associated with sleep disturbances such as insomnia and sleep fragmentation (Spiegelhalder et al., 2013). While originally thought of as side effects of the primary disorder, sleep disturbance is increasingly seen as integral component of many disorders. For example, specifically treating sleep disorders in individuals with major depression helps alleviate depressive symptoms (Sánchez-Ortuño and Edinger, 2012; Spiegelhalder et al., 2013). Furthermore, given the importance of sleep related memory consolidation and synaptic homeostasis as described here, it is easy to see how sleep disturbance could contribute to cognitive deficits.

Thus, we speculate that a contributing factor to the widespread occurrence of olfactory disorders, particularly odor identification, across diverse pathologies may be related to underlying sleep disorders. Memory and neural plasticity are integral not only to odor memory, but also to basic odor perception (Wilson and Stevenson, 2003; Wilson and Sullivan, 2011). As described here and elsewhere, sleep, especially slow-wave sleep, is now known to play an important role in that plasticity, including modulation of synaptic connectivity (Tsuno et al., 2008) and survival (Yokoyama et al., 2011) of OB neurons known to be critical for precise odor discrimination (Gheusi et al., 2000; Moreno et al., 2009). Furthermore, disruption of normal sleep-related activity within the olfactory cortex can impair the strength and accuracy of odor memory, leading to impaired odor-guided behavior (Barnes and Wilson, 2014). In addition, 24 h of sleep deprivation has been shown to impair odor identification in humans (Killgore and McBride, 2006). Thus, olfactory perception and memory may benefit from a good night’s sleep.
